# The Cancer Cure

**Published:** 1872-04

**Authors:** 


### THE CANCER CURE.

THE Cundurango Bark furnished through the British Government to two of the large London hospitals, is reported by the physicians who used it to be absolutely worthless as a remedy for cancer. It is said that there are two kinds, the genuine and the spurious.

Some years ago the newspapers were paid to announce that sarsaparilla was prepared by a house in New York with a peculiar machinery and a new manipulation, by which the acknowledged virtues of the plant were obtained in all their strength. The machine ran, made the druggists rich before the people could find out the truth of the matter, and now we do not remember to have seen sarsaparilla, prepared by this peculiar apparatus, advertised in a year. There is no doubt that sarsaparilla is as good now as it ever was — for nothing.

Then another man came along, who had a preparation of "bark" and iron; his theory was that iron was an invaluable remedy, but that it was so heavy that it would sink to the bottom in any liquid employed for its use; but his "bark" preparation had the very peculiar quality of holding in solution precisely the amount needed to have the proper healthful effect. He had ever so many certificates from Boston, and with true Yankee pertinacity he "pushed things" for two years, honestly believing that his preparation of iron was the best ever known since that fellow used it, who lived before the flood, in making harps and organs. But, poor man! he worked so hard in talking up his medicine that it killed him. And who now hears of the iron which don't sink in water?

Millions of people have died while waiting for the cure which the medicine was to effect, which they had been taking so industriously for weeks and months. All of us remember the wonderful cures effected by various "panaceas," while the panaceas themselves have passed from public notice, either because all the people were cured or the medicine was worthless.

This brings us back to first principles. If you are sick, consult a physician who has necessarily a daily experience in the observation and treatment of ailments like your own, and whose reputation and pecuniary interests strongly prompt him to do all he can to cure you.

If you were travelling through a wilderness country, to which you were an entire stranger, and were anxious to reach your journey's end as soon as possible, without losing time by taking the wrong road, you would gladly avail yourself of the opportunity of securing a guide who had travelled along that way several times. Tom Fool says, "O, I don't want your advice; I know better than you."

